# Rice Protein Reduces Triglyceride Levels through Modulating CD36, MTP, FATP, and FABP Expression in Growing and Adult Rats

**DOI:** 10.3390/foods13172704

**Published:** 2024-08-27

**Authors:** Bingxiao Liu, Zhengxuan Wang, Mingcai Liang, Lin Yang

**Affiliations:** School of Chemistry and Chemical Engineering, Harbin Institute of Technology, Harbin 150001, China; m18845618140@163.com (B.L.); 18646199358@163.com (Z.W.); lmc6677@163.com (M.L.)

**Keywords:** triglyceride-lowering action, triglyceride transport, free fatty acid, age

## Abstract

To elucidate the effect of rice protein on the regulation of triglyceride transport to reduce triglyceride levels, growing and adult male Wistar rats were fed with casein and rice protein for 2 weeks. With the intake of rice protein, the gene and protein expressions of cluster determinant 36 (CD36), microsomal triglyceride transfer protein (MTP), fatty acid transport protein-2 (FATP-2), and fatty acid-binding protein-1 (FABP-1) were, respectively, downregulated in growing and adult rats, suggesting rice protein could effectively regulate triglyceride transport. As a result, rice protein significantly reduced plasma levels of triglyceride and fatty acids, while hepatic accumulations of triglyceride and fatty acids were also decreased via rice protein. The present study demonstrates that RP exerts regulatory effects on CD36, MTP, FATP-2, and FABP-1 expression in growing and adult rats, revealing a link to triglyceride-lowering actions and the modulations of triglyceride transport exerted by rice protein. Results suggest that the aging process cannot attenuate the depression of CD36, MTP, FATP, and FABP 19 expression to reduce triglyceride levels induced by rice protein.

## 1. Introduction

Rice (*Oryza sativa*) is a widely cultivated major cereal crop worldwide [1,2]. Rice is a primary staple food in Asian countries, providing nutritional sustenance to over 2 billion people in the region, and China is the largest producer of rice in the world. As we all know, rice protein (RP) is a major plant protein [3,4]. And rice protein exerts various physiological benefits to prevent some metabolic diseases, such as hyperlipidemia, diabetes, obesity, etc. [5,6,7,8]. Similar to soy protein [9,10], rice protein also has a lipid-lowering effect and can reduce body weight. Our previous studies indicated that rice protein could reduce triglyceride levels [6,7]. Moreover, it has been demonstrated that rice protein could upregulate lipolysis and downregulate lipogenesis to inhibit triglyceride accumulation in growing rats [7]. Further, the depressions of hepatic total- and very low-density lipoprotein (VLDL)-triglyceride output by rice protein have been corroborated in the perfusion study, suggesting that rice protein could suppress triglyceride secretion into the circulation [11]. However, up to now, the precise molecular mechanism by which rice protein regulates triglyceride transport involving triglyceride-lowering action is not fully established.

Triglyceride transport is a complex process, which can be controlled by various regulators, including the cluster determinant 36 (CD36), microsomal triglyceride transfer protein (MTP), fatty acid transport protein (FATP), fatty acid-binding protein (FABP), etc. [12,13,14,15,16]. As a class B scavenger receptor and fatty acid translocase, CD36 serves as a facilitator of lipid transport and mediates the uptake of various lipids, e.g., long-chain fatty acid (LCFA) and oxidized low-density lipoprotein (ox-LDL) [12,17,18,19,20]. MTP exerts triacylglycerol transfer activity and VLDL secretion [13,14,21,22]. FATP and FABP are key actors for fatty acid transport and uptake [19,23,24,25,26]. Thus, to elucidate the triglyceride transport mechanism involved in triglyceride-lowering action, the expressions of CD36, MTP, FATP, and FABP should be particularly taken into account.

Lipid metabolism can be affected by age [27]. Thus, growing and adult rats were used in this study. The key questions addressed are the following: (1) is there a link between the triglyceride-lowering action exerted by rice protein and the modulations of triglyceride transport in growing and adult rats? (2) Does age affect the regulatory effect of rice protein on CD36, MTP, FATP, and FABP expression?

## 2. Materials and Methods

### 2.1. Animals Experiments

As the same with our previous studies, rice protein and casein (CAS) were used as dietary protein sources [28,29,30,31]. Consistent with the approach described in our previous study [7,32], rice protein was extracted from *Oryza sativa* L. cv. *Longjing* 20 (Rice Research Institute of Heilongjiang Academy of Agricultural Sciences, Jiamusi, China) via the alkaline method. Casein, as a control, was purchased from Gansu Hualing Industrial Group (Gansu, China). According to the formula recommended by the American Institute of Nutrition (AIN-93), animal diets were prepared for growing and adult rats [33].

In this study, growing male Wistar rats (body weight 200–220 g, purchased from the Vital River Laboratory Animal Technology Co., Ltd., Beijing, China) were fed ad libitum casein (CAS-G) and rice protein (RP-G) with a dietary protein level of 20% (as crude protein, CP, according to AIN-93G) for 2 weeks, while adult male Wistar rats (body weight 380–400 g, purchased from the Vital River Laboratory Animal Technology Co. Ltd.) were fed ad libitum 14% (as CP, according to AIN-93M) dietary protein from casein (CAS-A) and rice protein (RP-A) for 2 weeks.

Animal experiments were approved and performed in conformity with the Guidelines of the Committee for the Experimental Animals of the Harbin Institute of Technology (IACUC-2020009, Harbin, China). Growing and adult rats were individually housed in metabolic cages in a room maintained at 22 ± 2 °C under a 12 h light–dark cycle (07:00–19:00 for light). All rats were allowed free access to commercial pellets (Vital River Laboratories, Beijing, China) for 3 days. After acclimatization, rats were randomly divided into four experimental groups (CAS-G, RP-G, CAS-A, and RP-A). Each group consisted of six animals with similar body weight.

Consistent with our previous studies [28,29,30,31], food intake and body weight were recorded daily in the morning before replenishing the diet during the feeding period. At the end of the feeding period, the rats were deprived for 12 h and then sacrificed. Blood was withdrawn from the abdominal vein into a heparinized syringe under anesthesia with sodium pentobarbital (50 mg/kg body weight), immediately cooled on ice, and separated via centrifugation at 12,000× *g* for 5 min. The plasma obtained was frozen at −20 °C until analysis. After blood collection, the liver was quickly freeze-clamped in liquid nitrogen and stored at −80 °C until analysis.

### 2.2. Measurement of Triglyceride and Free Fatty Acid Contents

The contents of triglyceride and free fatty acids in the plasma and liver were measured with the commercial kits (Nanjing Jiancheng Bioengineering Institute, Nanjing, China).

### 2.3. Quantitative Real-Time PCR

Quantitative real-time PCR was performed according to previous studies [34,35,36]. Total RNA was extracted from livers of growing and adult rats after 2 weeks feeding, using the TRIzol reagent kit (Invitrogen, Carlsbad, CA, USA) according to the manufacturer’s protocol. cDNA was reverse transcribed from 1 g of total RNA using a PrimeScript™ 1st strand cDNA Synthesis Kit (Takara Bio. Inc., Otsu, Shiga, Japan). For quantitative real time PCR, cDNA was analyzed with the ABI 7500 sequence detection system (Applied Biosystems, Foster City, CA, USA) using SYBR Green (Takara Bio. Inc., Otsu, Shiga, Japan). The results were normalized to the level of glyceraldehyde-3-phosphate dehydrogenase (GAPDH) mRNA. The primer sequences used are shown in Table 1. In this study, the relative mRNA level in group CAS-G and CAS-A was, respectively, set as 1.00.

### 2.4. Western Blotting Analysis

As described in our previous studies [30,31], hepatic proteins were extracted and used for Western blot analysis. The proteins with the 2 × SDS sample loading buffer were denatured via boiling for 5 min and were separated using 10% SDS-PAGE. The gel was transferred onto polyvinylidene difluoride (PVDF) membrane (Millipore, Bedford, MA, USA). After blocking with 5% fat-free milk in TBS at room temperature for 1 h, the membranes were incubated overnight at 4 °C with primary antibodies for CD36 (Santa Cruz Biotechnology, Santa Cruz, CA, USA), MTP (Santa Cruz Biotechnology), FATP-2 (Proteintech, Wuhan, China), FABP-1 (Proteintech), β-actin (Cell Signaling, Danvers, MA, USA), and GAPDH (Proteintech). Next, the membranes were washed with TBST (TBS with 0.1% Tween-20) three times and incubated at room temperature for 2 h with a second antibody (Santa Cruz Biotechnology). The protein bands were visualized using ECL reagent (Beyotime, Shanghai, China). The amount of protein was quantified using QuantityOne 4.6.2 software (Bio-Rad, Hercules, CA, USA). In this study, the relative protein expression in group CAS-G and CAS-A was, respectively, set as 1.00.

### 2.5. Statistical Analysis

Data are expressed as the mean ± SEM. Differences between groups were examined for statistical significance using one-way analysis of variance (ANOVA) followed by the least significant difference test. The criterion for significance was *p* < 0.05.

## 3. Results and Discussion

### 3.1. The Chemical Analysis of the Extracted Rice Protein

As shown in Table 2, the rice protein extracted in this study contained 90.76% protein, with non-protein components accounting for only 9.24%. Water was the second-largest component, accounting for approximately 6.61%. Besides water, the primary non-protein components included starch, lipid, ash, and crude fiber, which together constituted about 2.63% of the total composition. Given the extremely low levels of starch, lipid, ash, and crude fiber in the extracted rice protein, the potential interference from these components on the experimental results is considered negligible.

### 3.2. Body Weight Gain and Food Intake

**Figure 1:**
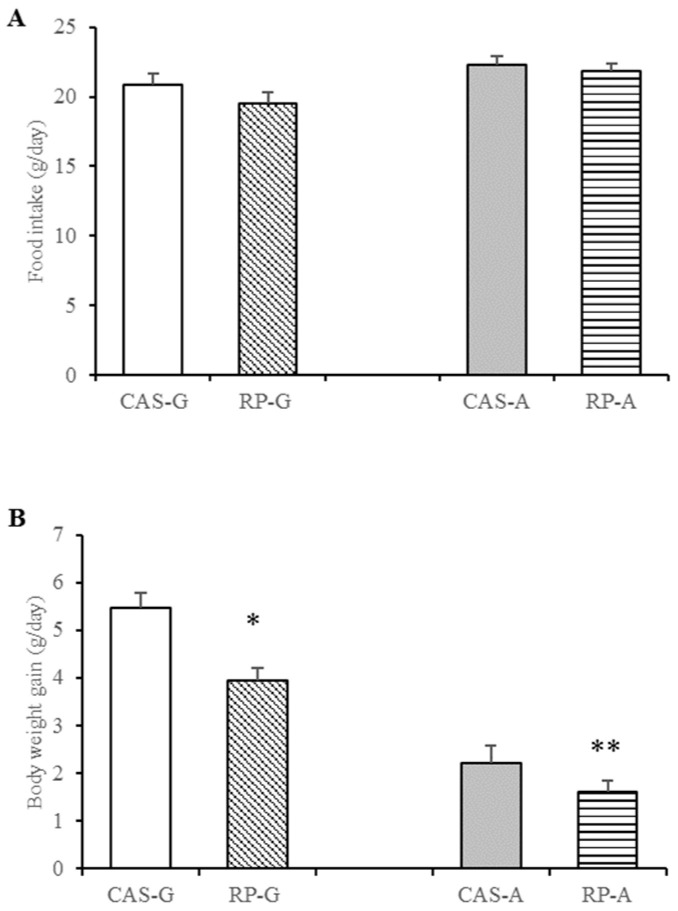
Food intake and body weight gain of growing and adult rats. (**A**) Food intake of growing and adult rats. (**B**) Body weight gains of growing and adult rats. Values are the means ± SEM (*n* = 6). * *p* < 0.05, in comparison with CAS-G. ** *p* < 0.05, in comparison with CAS-A. CAS-A, adult rats fed with casein for 2 weeks; CAS-G, growing rats fed with casein for 2 weeks; RP-A, adult rats fed with rice protein for 2 weeks; RP-G, growing rats fed with rice protein for 2 weeks.

As shown in Figure 1, after 2 weeks feeding, although food intakes were not significantly different between the CAS and RP groups (*p* > 0.05, Figure 1A), body weight gains were, respectively, reduced for the RP-G group by 27.66% (*p* < 0.05, Figure 1B) and for the RP-A group by 27.60% (*p* < 0.05, Figure 1B), as compared with the CAS-G and CAS-A groups.

Thus, we can see that there was no significant difference in the food intake of rats in the CAS and RP groups during the two-week feeding period. However, surprisingly, the body weights of the rats in the RP group were generally lower than those in the CAS group. This amazing result was presented in both growing and adult rats. The result fully demonstrates that rice protein can effectively reduce the body weight of rats and lower the risk of obesity. And this effect is not constrained by the influence of the age of experimental animals. This finding is similar to the reported results in previous studies [9,31,37,38]. Therefore, we next explored the effect of rice protein on triglyceride levels.

### 3.3. Effect of Rice Protein on Triglyceride Levels

**Figure 2:**
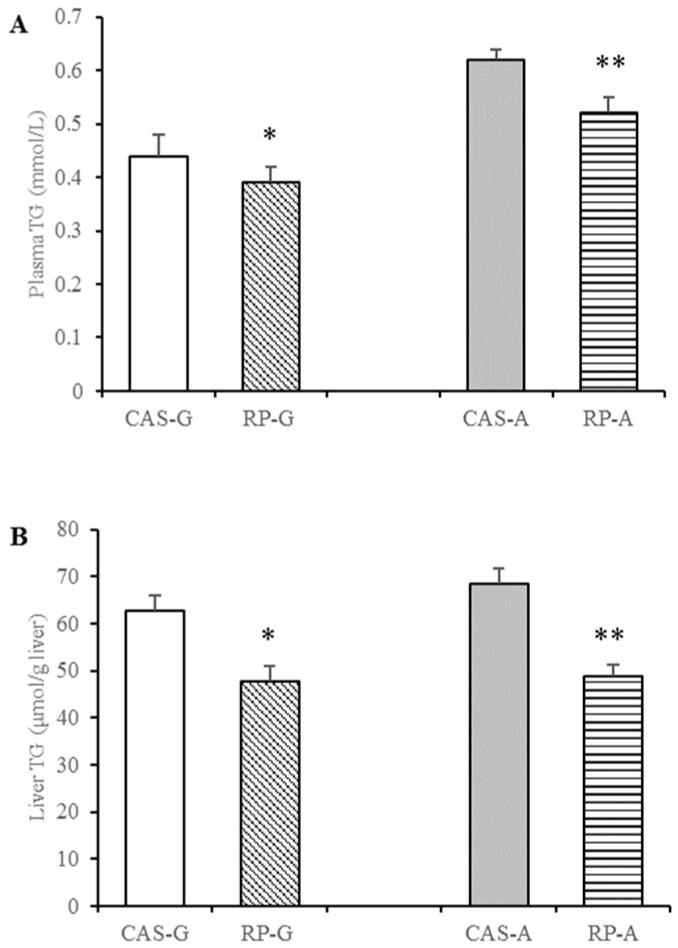
Plasma and liver triglyceride levels in growing and adult rats. (**A**) Plasma triglyceride levels in growing and adult rats. (**B**) Liver triglyceride levels in growing and adult rats. Values are the means ± SEM (*n* = 6). * *p* < 0.05, in comparison with CAS-G. ** *p* < 0.05, in comparison with CAS-A. CAS-A, adult rats fed with casein for 2 weeks; CAS-G, growing rats fed with casein for 2 weeks; RP-A, adult rats fed with rice protein for 2 weeks; RP-G, growing rats fed with rice protein for 2 weeks; TG, triglyceride.

As illustrated in Figure 2, after 2 weeks feeding, compared with CAS-G and CAS-A, plasma triglyceride levels were distinctly depressed in the RP-G group by 11.36% and in the RP-A group by 16.13% (*p* < 0.05, Figure 2A). Similarly, hepatic triglyceride accumulations were also distinctly depressed in the RP-G group by 23.99% and in the RP-A group by 28.78% (*p* < 0.05, Figure 2B), as, respectively, compared to the CAS-G and CAS-A groups.

Thus, after 2 weeks feeding, the triglyceride-lowering action exerted by rice proteins emerged from this study, in agreement with our previous findings [6]. This finding is also consistent with the results reported in [9,39,40]. The results suggested that rice protein could reduce triglyceride levels in growing and adult rats, independent of the influence of age.

### 3.4. Effect of Rice Protein on Free Fatty Acids Content

**Figure 3:**
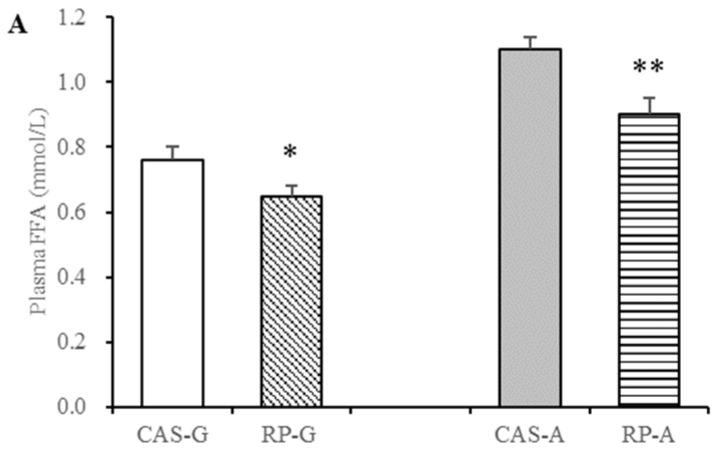

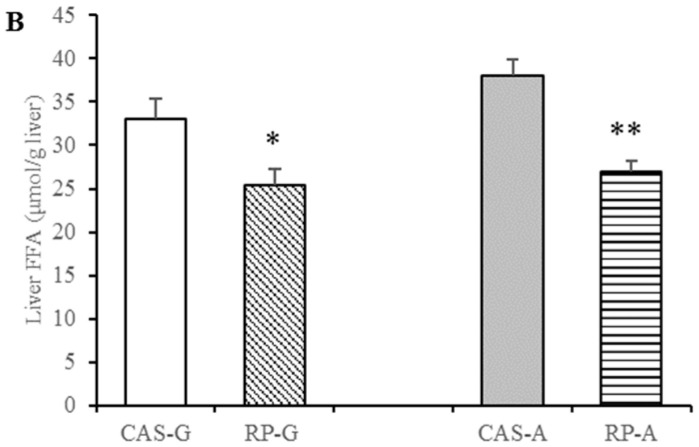
Plasma and liver free fatty acid contents in growing and adult rats. (**A**) Plasma free fatty acid contents in growing and adult rats. (**B**) Liver free fatty acid contents in growing and adult rats. Values are the means ± SEM (*n* = 6). * *p* < 0.05, in comparison with CAS-G. ** *p* < 0.05, in comparison with CAS-A. CAS-A, adult rats fed with casein for 2 weeks; CAS-G, growing rats fed with casein for 2 weeks; FFA, free fatty acid; RP-A, adult rats fed with rice protein for 2 weeks; RP-G, growing rats fed with rice protein for 2 weeks.

After rice protein intake for 2 weeks, the downward trend in free fatty acid contents were also observed in the rice protein groups, a similar trend to the triglyceride levels. Compared with CAS-G, RP-G exhibited significantly decreased free fatty acid contents to the degrees of 14.47% less in the plasma (*p* < 0.05, Figure 2A) and of 23.29% less in the liver (*p* < 0.05, Figure 3B). As for adult rats, free fatty acid contents were markedly decreased in the RP-A group by 18.18% in the plasma (*p* < 0.05, Figure 3A) and 29.01% in the liver (*p* < 0.05, Figure 3B).

In this study, the decreased levels of free fatty acids in the RP-G and RP-A groups further suggested that rice protein could exert effective lowering effects on triglyceride accumulation in growing and adult periods, despite the influence of age. This finding further validates our previous findings [7,37].

### 3.5. Effect of Rice Protein on CD36 Expression

In order to elucidate the triglyceride-lowering action of rice protein, the triglyceride transport mechanism exerted by rice protein was investigated in this study.

**Figure 4:**
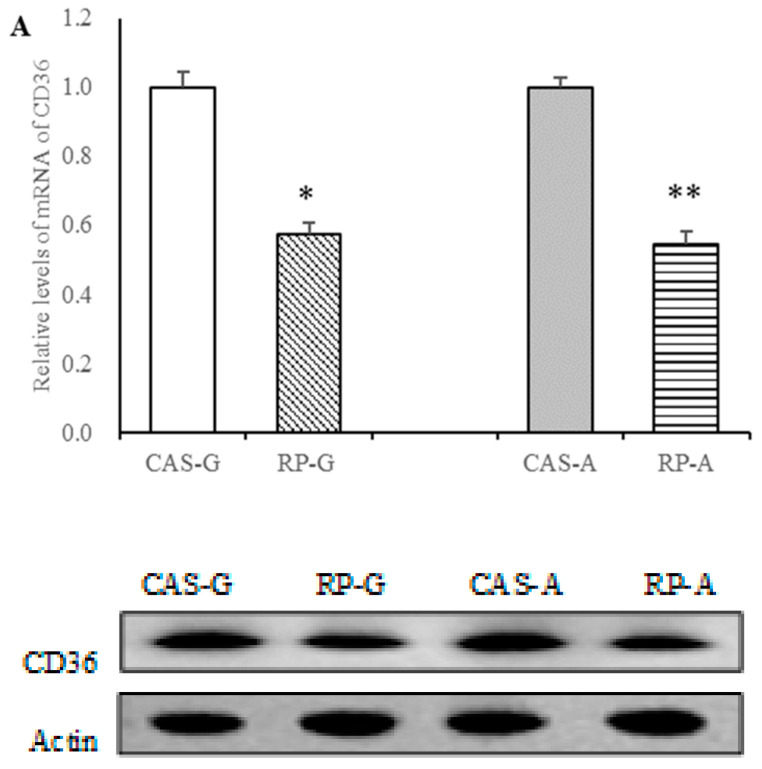

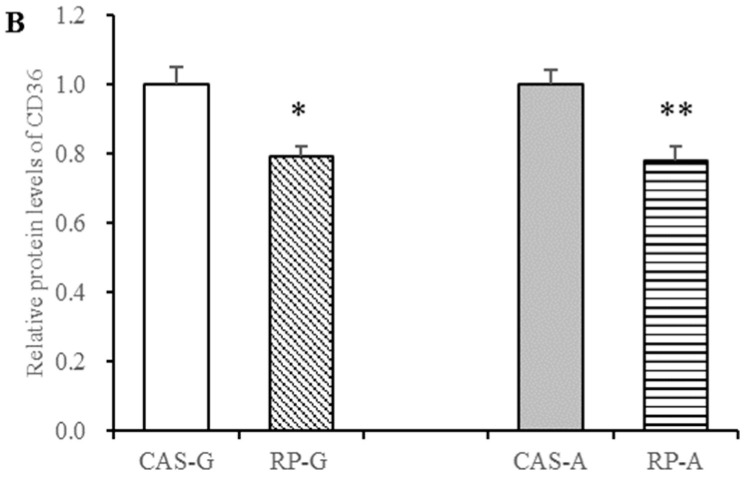
Hepatic mRNA levels and protein expression of CD36 in growing and adult rats. (**A**) Hepatic mRNA levels of CD36. (**B**) Hepatic protein expressions of CD36. Values are the means ± SEM (*n* = 6). * *p* < 0.05, in comparison with CAS-G. ** *p* < 0.05, in comparison with CAS-A. CAS-A, adult rats fed with casein for 2 weeks; CAS-G, growing rats fed with casein for 2 weeks; CD36, cluster determinant 36; RP-A, adult rats fed with rice protein for 2 weeks; RP-G, growing rats fed with rice protein for 2 weeks.

Firstly, we measured CD36 expressions. As shown in Figure 4, after 2 weeks feeding, rice protein effectively depressed protein and gene expressions of CD36 in growing and adult rats. Compared with the CAS-G and CAS-A groups, the mRNA levels of CD36 were markedly decreased in the RP-G and RP-A groups (*p* < 0.05, Figure 4A). Similarly, the RP-G and RP-A groups exhibited lower protein expression of CD36 in growing and adult rats. As illustrated in Figure 4B, hepatic protein levels of CD36 were dramatically reduced by rice proteins, accounting for decreases of 20.61% in the RP-G group (*p* < 0.05) and of 21.97% in the RP-A group (*p* < 0.05), as compared to CAS-G and CAS-A. Results showed that CD36 expressions could be downregulated by rice protein feedings in growing and adult rats, for which no significant differences in gene and protein expressions were observed between the RP-G and RP-A groups (*p* > 0.05), suggesting that the increased age did not attenuate the suppression of CD36 expression induced by rice protein.

CD36 is an important lipid transporter for fatty acid uptake [17,18,19]. CD36 expression and fatty acid uptake are linked to some diseases, such as hyperlipidemia, obesity, etc. [12,18]. It is clear that the increases in hepatic fatty acid uptake and triglyceride accumulation are attributed to the stimulation of liver CD36 expression, whereas the absence of CD36 can protect fatty acid uptake and reduce triglyceride levels [41,42,43,44]. Thus, CD36 plays a major role in fatty acid uptake to regulate triglyceride levels. In this study, we confirm and expand this view. Results showed a significant positive correlation between the expression of CD36 and the concentration of liver fatty acid content (r = 0.7987, *p* < 0.05), as well the accumulation of liver triglycerides (r = 0.8519, *p* < 0.05), suggesting that rice protein could depress CD36 expression to reduce fatty acid uptake and triglyceride accumulation.

In addition to the uptake of fatty acid, CD36 also governs triglyceride levels through regulating triglyceride secretion [12,43]. Some studies have demonstrated that CD36 deletion can suppress VLDL output to reduce triglyceride secretion, suggesting that the absence of CD36 can reduce triglyceride levels [44,45]. Combined with our previous findings that the triglyceride-lowering action of rice protein is attributed to the suppression of hepatic VLDL-triglyceride secretion [11], the current study clearly reveals an insight that the decreased CD36 expressions as a result of rice protein might be linked with the triglyceride-lowering action of rice protein. To support this view, a significant positive correlation between the expression of CD36 and plasma triglyceride levels (r = 0.7934, *p* < 0.05) was found in this study. Thus, the triglyceride-lowering mechanism exerted by rice protein might be ascribed in part to the suppression of CD36 expression, which was pivotal for governing fatty acid uptake and triglyceride secretion.

### 3.6. Effect of Rice Protein on MTP Expression

MTP is another important regulator for triglyceride transport [13,14]. Thus, in this study, the regulatory effect of rice protein on MTP expression was also determined after 2 weeks feeding.

**Figure 5:**
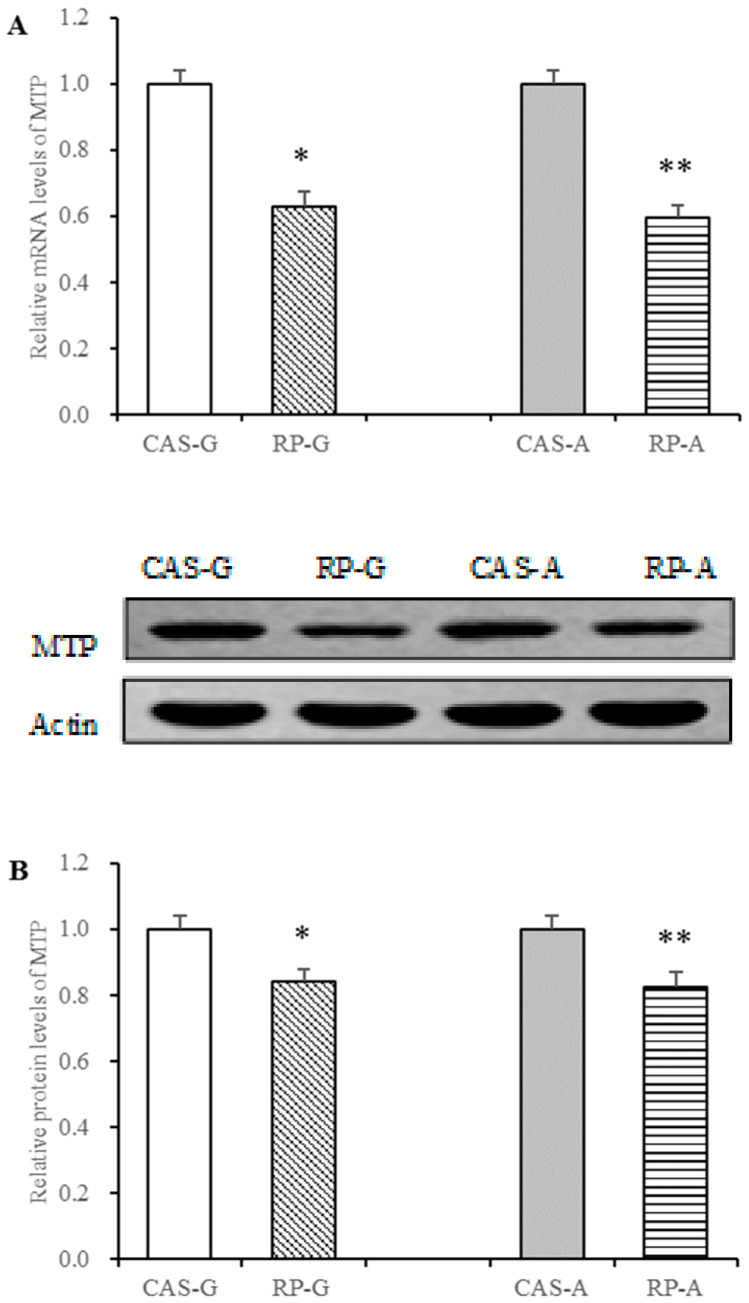
Hepatic mRNA levels and protein expression of MTP in growing and adult rats. (**A**) Hepatic mRNA levels of MTP. (**B**) Hepatic protein expression of MTP. Values are the means ± SEM (*n* = 6). * *p* < 0.05, in comparison with CAS-G. ** *p* < 0.05, in comparison with CAS-A. CAS-A, adult rats fed with casein for 2 weeks; CAS-G, growing rats fed with casein for 2 weeks; MTP, microsomal triglyceride transfer protein; RP-A, adult rats fed with rice protein for 2 weeks; RP-G, growing rats fed with rice protein for 2 weeks.

With the intake of rice protein, the dramatic decreases in the mRNA levels of MTP were, respectively, exhibited in the RP-G and RP-A groups (*p* < 0.05, Figure 5A). Compared with the CAS-G and CAS-A groups, the protein levels of MTP were also significantly decreased in the RP-G group by 16.06% and in the RP-A group by 17.37% (*p* < 0.05, Figure 5B).

MTP is an essential transporter for the assembly and export of triglyceride-rich VLDL, serving as a facilitator of triglyceride transfer [21,22]. Some studies indicated that hepatic overexpression of MTP leads to increased secretion of VLDL-triglycerides, whereas the inhibition of MTP results in decreased triglyceride secretion, suggesting that MTP is rate-limiting for VLDL-triglyceride secretion [46,47]. Previously, we have demonstrated that rice protein significantly depressed the output of VLDL-triglyceride in a perfusion study [11]. Thus, the current observation that rice protein could suppress MTP expression strongly supports our previous findings, suggesting that the suppression of MTP expressions by rice protein might be a contributor to the decreased output of VLDL-triglyceride involved with the triglyceride-lowing action of rice protein.

MTP not only improves VLDL export, but also accelerates the transport of triglycerides. Thus, the inhibition of MTP is preferable to reduced plasma levels of triglyceride. It is clear that hypertriglyceridemia is associated with increased MTP expression, whereas the downregulation of liver MTP is a therapeutic intervention to lower plasma triglyceride levels, suggesting that a novel feasible anti-hypertriglyceridemic mechanism is to decrease liver MTP [48,49]. In this study, the results that rice protein effectively suppressed the expressions of hepatic MTP and significantly reduced plasma triglyceride levels confirm and support this view, showing a significant positive correlation between the expression of MTP and plasma triglyceride levels (r = 0.8301, *p* < 0.05).

Moreover, a significant positive correlation between the expression of MTP and hepatic triglyceride contents (r = 0.8143, *p* < 0.05) was also found in this study. This finding might raise the question of why the suppression of MTP expression for attenuating triglyceride transport did not cause excessive hepatic triglyceride accumulation in the rice protein groups. To explain this interesting finding, the view that lipogenesis and lipolysis are two major processes for hepatic triglyceride storage should be taken into account [5]. It has been demonstrated that rice protein can reduce hepatic activities of fatty acid synthase (FAS), glucose 6-phosphate dehydrogenase (G6PD), and malate dehydrogenase (MDH), whereas the activities of lipoprotein lipase (PL) and hepatic lipase (HL) can be stimulated by rice protein, suggesting that rice protein can inhibit lipogenesis and stimulate lipolysis [7]. Accordingly, it is convincing that the suppression of MTP expression for attenuating triglyceride transport did not cause excessive hepatic triglyceride accumulation in the rice protein group might be attributed to the fact that rice protein upregulates lipolysis and downregulates lipogenesis to result in less hepatic triglyceride contents in the liver.

Taken together, it is not unlikely that rice protein can reduce triglyceride levels not only in the plasma but also in the liver. The fact that rice protein can downregulate MTP expression and reduce triglyceride levels points towards the suppression of MTP expression as another important mechanism for the triglyceride-lowering action exerted by rice protein.

### 3.7. Effect of Rice Protein on FATP Expression

Triglyceride levels depend on fatty acid uptake, triglyceride synthesis, triglyceride catabolism, etc. Our previous studies have demonstrated that rice protein could decrease triglyceride synthesis and increase triglyceride catabolism [7]. Thus, to further elucidate the triglyceride-lowering mechanism, in this study, the regulation of fatty acid transport by rice protein was particularly emphasized through investigating FATP expression.

**Figure 6:**
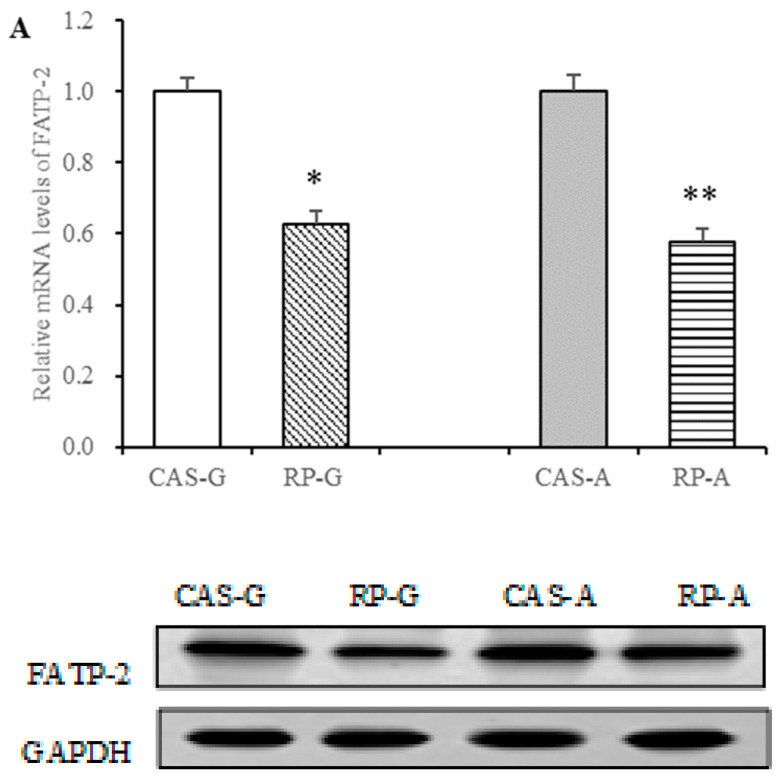

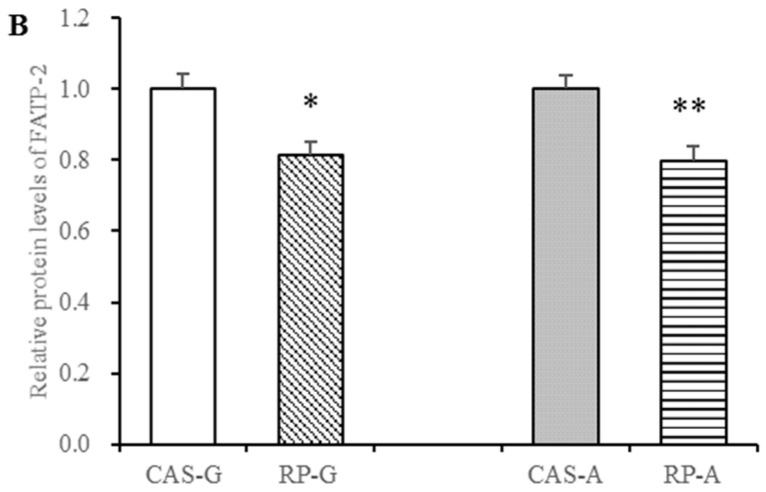
Hepatic mRNA levels and protein expression of FATP-2 in growing and adult rats. (**A**) Hepatic mRNA levels of FATP-2. (**B**) Hepatic protein expression of FATP-2. Values are the means ± SEM (*n* = 6). * *p* < 0.05, in comparison with CAS-G. ** *p* < 0.05, in comparison with CAS-A. CAS-A, adult rats fed with casein for 2 weeks; CAS-G, growing rats fed with casein for 2 weeks; FATP-2, fatty acid transport protein-2; RP-A, adult rats fed with rice protein for 2 weeks; RP-G, growing rats fed with rice protein for 2 weeks.

In this study, we focused on the expression of FATP-2 in the liver. Results showed that rice protein exhibited efficacious regulation of the expression of FATP-2 involved in fatty acid transport. As illustrated in Figure 6, the mRNA levels of FATP-2 were dramatically reduced via the intake of rice protein in growing and adult rats (*p* < 0.05, Figure 6A). Accompanied with a strong downregulation in gene expression, protein levels of FATP-2 were markedly depressed in the RP-G group by 18.68% and in the RP-A group by 20.19%, as compared to the CAS-G and CAS-A groups (*p* < 0.05, Figure 6B). Thus, it is evident that rice protein can exert a regulatory effect on fatty acid transport, in which the suppressed effect of rice protein on FATP-2 expression was not attenuated by increased age, showing that the suppression was not more efficacious in the RP-A group than in the RP-G group.

As an important fatty acid transporter, the major function of FATP is for fatty acid uptake [15,24]. In this study, rice protein significantly suppressed the expression of FATP-2, resulting in the reduced hepatic accumulations of fatty acids. To support this, there was a significant positive correlation between the expression of FATP-2 and the concentration of hepatic fatty acids (r = 0.8115, *p* < 0.05) in this study.

In addition to increasing the function for fatty acid uptake, FATP can promote triglyceride storage as well [15,24]. Thus, FATP is associated with triglyceride levels. To support this view, after 2 weeks feeding, accompanied with the suppressed FATP-2 expression, rice proteins significantly reduced triglyceride levels both in the plasma and in the liver. Results showed a significant positive correlation between the expression of FATP-2 and plasma triglyceride level (r = 0.8002, *p* < 0.05), as well the accumulation of hepatic triglyceride (r = 0.7849, *p* < 0.05).

Taken together, rice protein could attenuate fatty acid uptake, leading to reduced triglyceride storage, in which the suppression of FATP-2 by rice protein might be an important contributor to its triglyceride-lowering action.

### 3.8. Effect of Rice Protein on FABP Expression

Similar to the downregulation of FATP-2, the protein and gene expressions of FABP-1 in the liver were also depressed via rice protein feeding.

**Figure 7:**
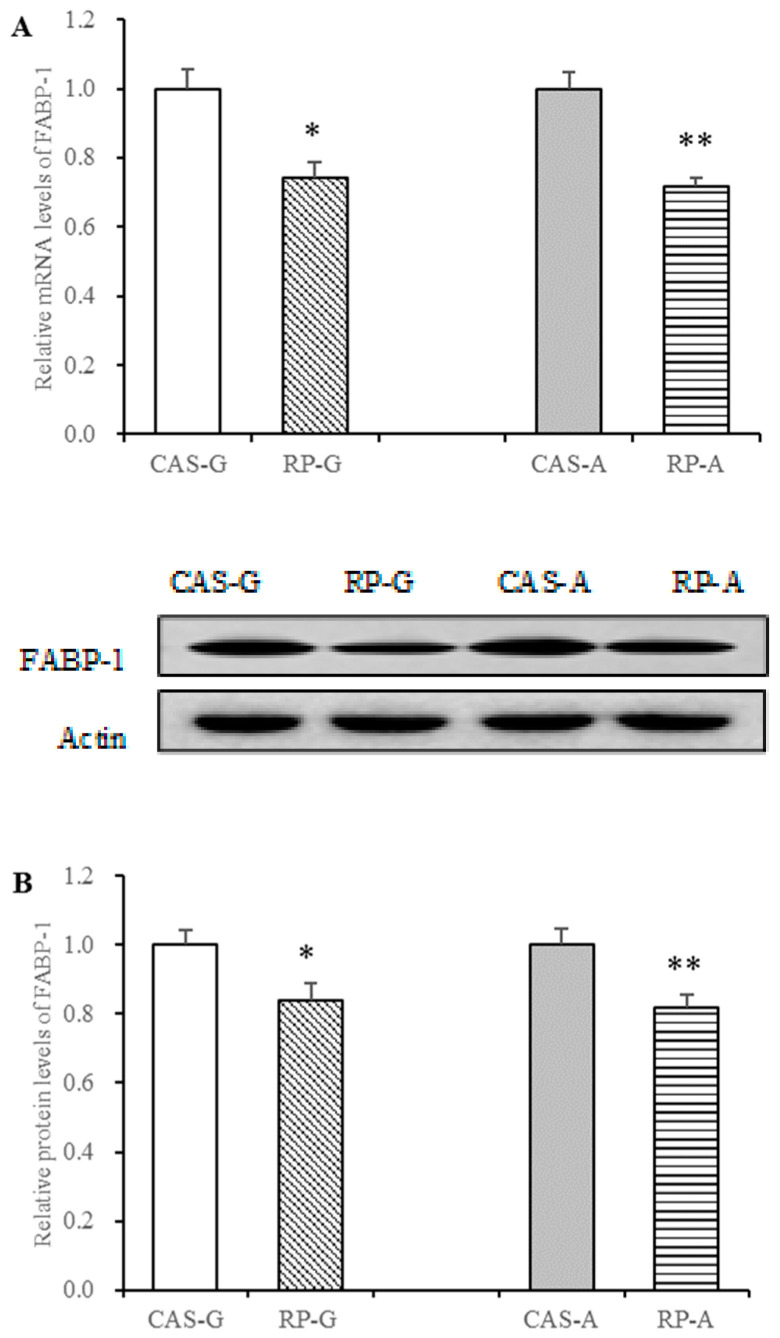
Hepatic mRNA levels and protein expression of FABP-1 in growing and adult rats. (**A**) Hepatic mRNA levels of FABP-1. (**B**) Hepatic protein expression of FABP-1. Values are the means ± SEM (*n* = 6). * *p* < 0.05, in comparison with CAS-G. ** *p* < 0.05, in comparison with CAS-A. CAS-A, adult rats fed with casein for 2 weeks; CAS-G, growing rats fed with casein for 2 weeks; FABP-1, fatty acid-binding-1; RP-A, adult rats fed with rice protein for 2 weeks; RP-G, growing rats fed with rice protein for 2 weeks.

With the intake of rice protein, the mRNA levels of FABP-1 were significantly decreased in the RP-G and RP-A groups with respect to the CAS-G and CAS-A groups (*p* < 0.05, Figure 7A). With a remarkable reduction in mRNA levels, the RP-G and RP-A groups also exhibited dramatic depression in FABP-1 protein expression as compared to the CAS-G and CAS-A groups, accounting for decreases of 16.14% in the RP-G group and of 18.26% in the RP-A group, respectively (*p* < 0.05, Figure 7B). Overall, these results further suggested that rice protein in both the RP-G and RP-A groups exhibited regulatory capacities on fatty acid transport.

FABP can facilitate fatty acid uptake for triglyceride storage [16,19]. It has been demonstrated that FABP-1 overexpression enhances fatty acid uptake in the liver, whereas ablation of FABP-1 inhibits fatty acid uptake and reduces fatty acid transport [24,25,50]. To confirm and support this view, a significant positive correlation between the expression of FABP-1 and the concentration of hepatic fatty acids (r = 0.7969, *p* < 0.05) was found in this study. Further, some evidence indicated that elevated levels of hepatic triglycerides are associated with upregulated expression of FABP, whereas hepatic triglyceride content is reduced in fabp−/− mice [50,51,52]. Consistent with these facts, there was a significant positive correlation between the expression of FABP-1 and the hepatic concentration of triglycerides (r = 0.7790, *p* < 0.05) in this study.

In addition to enhancing regulating fatty acid uptake, FABP can stimulate VLDL secretion as well. It is clear that elevated VLDL secretion is associated with increased FABP-1. In contrast, fabp−/− mice exhibit decreased VLDL production [16,24,25,50,51,52]. In light of this view, there was a significant positive correlation between the expression of FABP-1 and plasma triglyceride levels (r = 0.7885, *p* < 0.05) in this study, further supporting our previous studies that showed rice protein could depress the output of hepatic VLDL-triglyceride [11], which was associated to the triglyceride-lowering action.

Taken together, the suppression of FABP-1 expression might be one of the triglyceride-lowing mechanisms exerted by rice protein, resulting in the reduced net fatty acid uptake both for triglyceride storage and for VLDL export.

## 4. Conclusions

The present study is the first to demonstrate the regulatory effect of rice protein on CD36, MTP, FATP, and FABP expressions in growing and adult rats. Results provide convincing evidence to confirm a link between the triglyceride-lowering action and the modulations of triglyceride transport exerted by rice protein. The novel finding observed in this study is that the regulation of triglyceride transport via depression of CD36, MTP, FATP, and FABP expression reducing triglyceride levels is not attenuated by increased age. Clearly, more detailed investigations in future studies are needed to explore the precise triglyceride transport mechanism exerted by rice protein to produce triglyceride-lowering actions.

Although China is a major producer of rice, the industry is predominantly focused on producing standard rice, with a relatively low proportion of refined rice processing. The utilization rate of by-products such as broken rice and rice bran generated during processing is also low, resulting in a significant gap compared to the level of refined rice processing industrialization achieved by grain and oil enterprises in some developed countries. This study investigates the lipid-lowering physiological functions of rice protein, providing a theoretical foundation for the production of functional rice protein foods that regulate lipid levels. It aims to extend the rice processing industry chain in China and offer new avenues for refined, deep, and precise rice processing, thereby enhancing the added value of rice.

## Figures and Tables

**Table 1:** Sequences of primers for quantitative real-time PCR.

Gene	Forward	Reverse
GAPDH	ACAGCAACAGGGTGGTGGAC	TTTGAGGGTGCAGCGAACTT
CD36	TGCTGCACGAGGAGGAGAATGG	CACAGCCAGGACAGCACCAATAAC
MTP	TTCTGCCTACACTGGCTACG	TCTCCTCTCCCTCATCTGGA
FATP-2	TGTGGCTCTGGCTGGGACTG	GTAGCAGAGACTTGGCACGGATG
FABP-1	CCAGAAAGGGAAGGACATCAAGGG	TGGTCTCCAGTTCGCACTCCTC

**Table 2:** The content of nutritional components in extracted rice protein.

Nutrient Composition	Content (%)
Water	6.61
Protein	90.76
Starch	0.83
Lipid	0.43
Ash	0.89
Crude Fiber	0.48

## Data Availability

The original contributions presented in the study are included in the article, further inquiries can be directed to the corresponding author.
